# Bioactive and Antibacterial Glass Powders Doped with Copper by Ion-Exchange in Aqueous Solutions

**DOI:** 10.3390/ma9060405

**Published:** 2016-05-24

**Authors:** Marta Miola, Enrica Verné

**Affiliations:** Applied Science and Technology Department, Politecnico di Torino, Corso Duca degli Abruzzi 24, 10129 Torino, Italy; enrica.verne@polito.it

**Keywords:** bioactive glasses, ion-exchange, copper, antibacterial

## Abstract

In this work, two bioactive glass powders (SBA2 and SBA3) were doped with Cu by means of the ion-exchange technique in aqueous solution. SBA2 glass was subjected to the ion-exchange process by using different Cu salts (copper(II) nitrate, chloride, acetate, and sulphate) and concentrations. Structural (X-ray diffraction-XRD), morphological (Scanning Electron Microscopy-SEM), and compositional (Energy Dispersion Spectrometry-EDS) analyses evidenced the formation of crystalline phases for glasses ion-exchanged in copper(II) nitrate and chloride solutions; while the ion-exchange in copper(II) acetate solutions lead to the incorporation of higher Cu amount than the ion-exchange in copper(II) sulphate solutions. For this reason, the antibacterial test (inhibition halo towards *S. aureus*) was performed on SBA2 powders ion-exchanged in copper(II) acetate solutions and evidenced a limited antibacterial effect. A second glass composition (SBA3) was developed to allow a greater incorporation of Cu in the glass surface; SBA3 powders were ion-exchanged in copper(II) acetate solutions (0.01 M and 0.05 M). Cu-doped SBA3 powders showed an amorphous structure; morphological analysis evidenced a rougher surface for Cu-doped powders in comparison to the undoped glass. EDS and X-ray photoelectron spectroscopy (XPS) confirmed the Cu introduction as Cu(II) ions. Bioactivity test in simulated body fluid (SBF) showed that Cu introduction did not alter the bioactive behaviour of the glass. Finally, inhibition halo test towards *S. aureus* evidenced a good antimicrobial effect for glass powders ion-exchanged in copper(II) acetate solutions 0.05 M.

## 1. Introduction

Bioactive glasses have been widely investigated in the orthopaedic and dental fields for their ability to chemically bond to living bone through a well-known process, which involves a rapid ion-exchange between glass and surrounding biological fluids, the formation of silica-rich layer, the incorporation of calcium and phosphates, and the crystallization of biologically active hydroxyapatite (HAp) on their surface [1,2,3,4]. The peculiar surface reactivity of bioactive glasses and their corresponding glass-ceramics has been object of investigation for many years, since several authors focalized their attention on the multi-step mechanism for the formation of the HAp layer, which was considered the critical stage for bone healing [1,2,3,4,5,6,7]. Today, bioactivity (*i.e.*, the ability to form strong bonds to bone) is considered a useful stage for bone regeneration, but not the critical one. Recent studies report that the release of ionic dissolution products (such as Si^4+^, Mg^2+^, Ca^2+^) from the bioactive glass surface has stimulating effects on bone formation, playing a key role in the early stages of the bone regeneration processes [8,9,10]. For this reason, bioactive glass surfaces can stimulate both intracellular and extracellular responses and, in turn can be actively mineralized *in vivo*. The *in vivo* colonization of osteogenic stem cells and the stimulation of angiogenesis seem to be promoted by contact with bioactive glasses [8]. For these reasons, a new promising research field has been recently proposed—*i.e.*, the “genetic design” of new bioactive glass formulations [11] by doping silicate and phosphate glasses with several active ions, including trace elements [12,13].

Recently, some authors explored the aptitude of bioactive glasses to stimulate and regenerate soft tissues [10,14,15], and they proved that several processes that control soft tissue regeneration in the early steps can be regulated by the release of ions from bioactive glasses. In this prospective, several bioactive glass compositions containing specific metallic ions (Sr, Cu, Ag, Zn, *etc.*, reported as “bioinorganics”) with therapeutic effects were investigated [9,16,17].

Among the explored therapeutic agents, Cu was initially studied for its ability to induce differentiation of mesenchymal cells towards the osteogenic lineage [18]. More recently, a significant amount of cellular Cu has been found in human endothelial cells during angiogenesis [19], and its ability to stimulate the proliferation of endothelial cells has been investigated [20]. Some authors evidenced the ability of copper ions to promote angiogenesis [21,22] in particular by synergistic stimulating effects when associated with angiogenic growth factor FGF-2 [23]. Moreover, copper is also well known for its antimicrobial properties [24,25].

Silica-based Cu-containing bioactive glasses were obtained mainly by sol–gel process [26,27,28,29], evidencing the ability to incorporate copper up to 10% mol; the traditional melt and quenching technique was also adopted both for silica-based bioactive glasses [30] and phosphate glasses [31,32], demonstrating that the glass degradation and the consequent release of copper ions significantly reduced the bacterial adhesion and proliferation.

Moreover, Wu *et al.*, synthesized Cu-containing mesoporous bioactive scaffolds, which promoted the osteogenic differentiation of human bone marrow stromal cells, enhanced hypoxia-like tissue reaction, and showed antibacterial properties towards *E. coli*, thus combining angiogenesis, osteogenesis and antibacterial properties [33].

The ion-exchange process was also used to dope the surface of glasses (mainly soda-lime glass and Schott BK7 glass) with copper ions [34,35,36,37,38]. To the authors’ knowledge, this process was always performed in molten salts using different Cu salts, such as CuCl_2_, CuCl, CuCl:KCl and CuSO_4_:Na_2_SO_4_, or vapour of CuCl:KCl, and it was not applied to bioactive glass compositions.

In the present paper, the possibility to use the ion-exchange technique in aqueous solution to introduce copper in the outer layer of two different silica-based bioactive glasses was investigated for the first time by examining the effect of the process and the Cu introduction on the structure, morphology, composition, bioactive, and antibacterial properties of the glasses.

## 2. Materials and Methods

### 2.1. SBA2 Synthesis and Characterization

A bioactive glass with the composition of (mol %) 48% SiO_2_, 18% Na_2_O, 30% CaO, 3% P_2_O_5_, 0.43% B_2_O_3_, 0.57% Al_2_O_3_ (SBA2) was produced by means of melt and quenching technique. Briefly, the reactants were mechanically mixed, melted in a platinum crucible at 1450 °C for 1 h. Subsequently, the melt was cooled in water at room temperature obtaining a frit, which was mechanically milled in a zirconia jar and sieved to a final grain size <20 μm.

The obtained glass powders were subjected to an ion-exchange process; this technique is a mass transfer driven by concentration gradients, in which ions present in the surface of the glass (usually monovalent ions) diffuse out of the glass surface and are replaced by different ions coming from a molten salt bath or an aqueous solution. In this work, the process was performed in aqueous solution containing different Cu salts in different concentrations, as shown in Table 1, in order to introduce copper ions in the glass network.

In all experiments, 1 g of glass powder was immersed in 20 mL of solution and maintained at 37 °C for 1 h at 150 rpm to allow the exchange of copper ions with modifier ions of the glass (Na^+^ and Ca^2+^). Then, the solution was removed, the powders were washed two times with bi-distilled water, filtered using disk filter paper, and dried at 60 °C for 12 h.

The obtained powders were characterized in terms of structure by means of X-ray diffraction (XRD—X’Pert Philips diffractometer, PANalytical, Eindhoven, The Netherlands) to evaluate the possible formation of crystalline phases, using the Bragg Brentano camera geometry and the Cu-Ka incident radiation. The obtained pattern was analysed with X’Pert High Score software (2.2b) and the PCPDFWIN database (http://pcpdfwin.updatestar.com/).

The morphology and composition of ion-exchanged glass powders were analysed by means of Scanning Electron Microscopy (SEM, QUANTA INSPECT 200, FEI, Eindhoven, The Netherlands) equipped with Energy Dispersion Spectrometry (EDSPV 9900, EDAX, Mahwah, NJ, USA) to estimate the influence of the ion-exchange process on the glass powders and to verify the presence of Cu.

On the basis of structural and morphological-compositional characterizations, SBA2 powders ion-exchanged in a solution 0.01 M of copper acetate was selected to verify the antibacterial effect of Cu-doped glass. In order to perform the antibacterial ability test, 200 mg of glass powders were weighed and pressed at 4 tons for 10 s in an automatic press (Graseby T-40, Specac, Kent, UK) to obtain pellets. The inhibition halo test (Kirby Bauer test) was performed in accordance with NCCLS normative [39] using a standard *Staphylococcus aureus* strain (ATCC 29213).

*S. aureus* was selected since it is one of the strains mainly involved in the development of infection [40] and since the Gram positive strains are generally more resistant to the effects of antibacterial agents [41].

Briefly, a 0.5 McFarland solution, containing approximately 1 × 10^8^ colony forming units (CFU)/mL, was prepared by dissolving some *S. aureus* colonies, grown on blood agar plate, in physiological solution; the turbidity of the solution was evaluated by optical instrument—Phoenix Spec BD McFarland (Becton, Dickinson and Company, Franklin Lakes, NJ, USA). This bacterial suspension was uniformly spread on Mueller Hinton agar plate (Becton, Dickinson and Company, Franklin Lakes, NJ, USA), glass pellets were placed in contact with the agar and incubated overnight at 37 °C. At the end of incubation, the inhibition zone was observed and measured.

### 2.2. SBA3 Synthesis and Characterization

On the basis of the results obtained from Cu-doped SBA2 characterizations—in particular the amount of Cu introduced without affecting the glass structure and the antibacterial test (as reported in the following sections)—a second glass composition (SBA3) was developed in order to allow a greater incorporation of Cu in the glass surface. The SBA3 presents the following molar composition: 48% SiO_2_, 26% Na_2_O, 22% CaO, 3% P_2_O_5_, 0.43% B_2_O_3_, 0.57% Al_2_O_3_. In particular, the amount of Na_2_O was increased in order to increase the quantity of mobile monovalent ions as well as to produce a more opened amorphous network and enhance the reactivity, thus promoting the exchange of all modifier ions with copper ions.

SBA3 powders were obtained by means of melting and quenching process: SBA3 precursors were melted in a platinum crucible at 1450 °C for 1 h and cooled in water to obtain a frit, which was mechanically milled in a zirconia jar and sieved to a final grain size <20 μm.

Considering the ion-exchange conditions that allowed the introduction of Cu in the SBA2 glass without the formation of crystalline phases (as reported in the following sections), SBA3 powders (1 g) were ion-exchanged in aqueous solution of copper acetate 0.01 M and 0.05 M (20 mL) for 1 h at 37 °C and 150 rpm. At the end of the process, the solution was removed and SBA3 powders were washed and dried following the same process used for SBA2 glass.

The structure of SBA3 and ion-exchanged SBA3 (named from now on SBA3-Cu0.01 and SBA3-Cu0.05) was investigated by means of XRD (X’Pert Philips diffractometer) using the Bragg Brentano camera geometry and the Cu-Ka incident radiation; the obtained patterns were analysed with X’Pert High Score software and the PCPDF data bank. SEM-EDS analyses were performed to estimate the morphology and the composition, in particular the effective Cu introduction, of the three glasses. The composition of the glasses and the oxidation state of Cu were also investigated by means of X-ray photoelectron spectroscopy (XPS—PHI 5000 VERSA PROBE, PHYSICAL ELECTRONICS). Survey analyses were carried out to verify the glasses’ compositions, and high resolution spectra of C and Cu were performed to individuate the oxidation state of copper.

The *in vitro* bioactivity of SBA3, SBA3-Cu0.01, and SBA3-Cu0.05 was evaluated by immersing 100 mg of glass powders in 100 mL of simulated body fluid (SBF-Kokubo [42]), maintained at 37 °C and 150 rpm for 1, 3, 7, 14, 21, and 28 days (two samples for each time period). The pH of the solutions was monitored every 2–3 days, and at the end of each incubation time the SBF solution was removed, samples gently washed with bi-distilled water and acetone, filtered using disk filter paper, and dried at 37 °C overnight. Subsequently, samples were analysed by means of XRD and Field Emission Scanning Electron Microscopy (FESEM, ZEISS, Jena, Germany) equipped with EDS to evaluate the precipitation of hydroxyapatite (HAp) and the eventual influence of Cu in the bioactivity process.

The antibacterial effect of Cu-doped glasses was investigated by means of the inhibition halo test previously described for SBA2 glass by using Cu-doped glass pellets, a standard *S. aureus* strain, and respecting the NCCLS normative [39].

## 3. Results and Discussion

### 3.1. SBA2 Characterization

**Figure 1:**
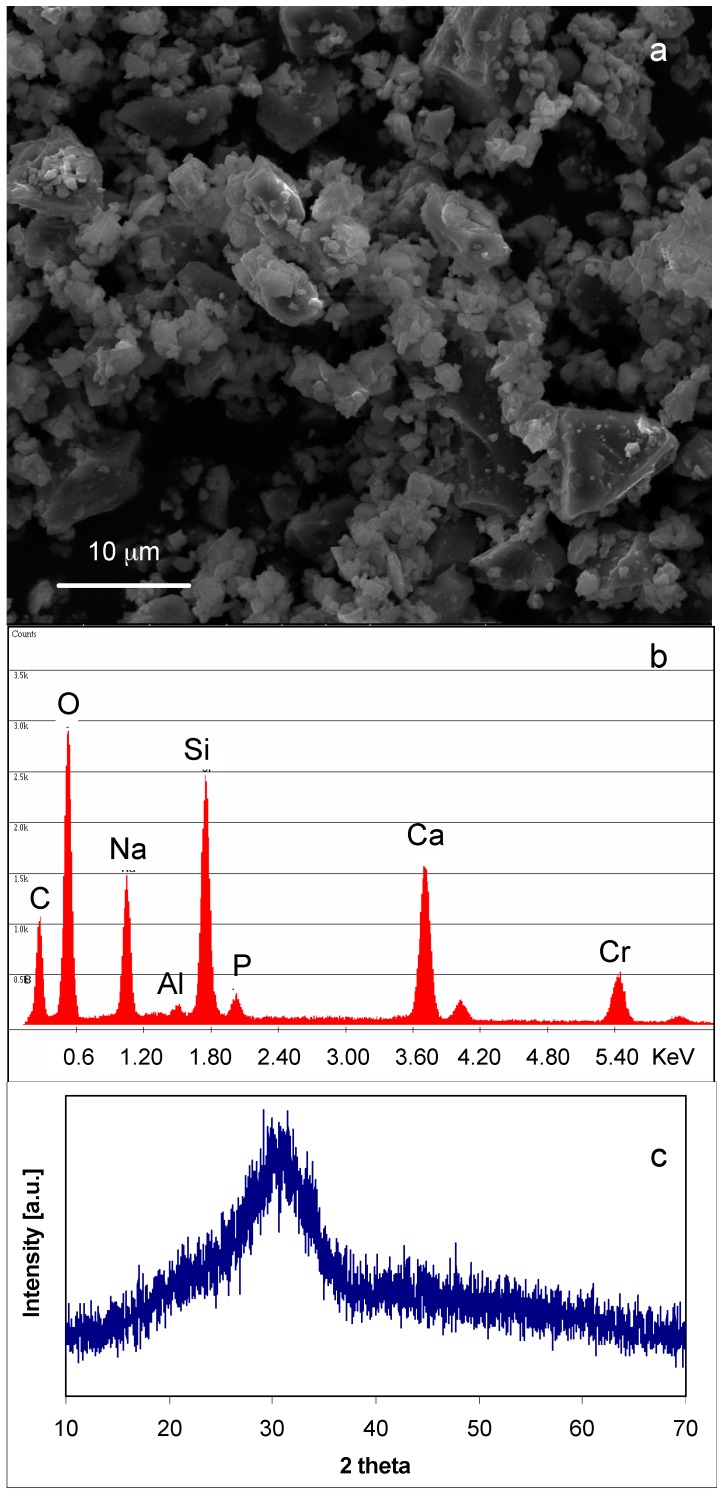
(**a**) Morphological; (**b**) compositional; and (**c**) structural analyses of SBA2 bioactive glass powders.

Figure 1 shows the morphological, compositional, and structural characterization of SBA2 powders. As can be observed (Figure 1a), the powders’ size is <20 μm and they have an irregular shape due to the milling process. EDS analysis (Figure 1b) confirmed the presence of all elements characteristics of the glass, except for boron—due to its low atomic weight, it was not detected by the instrument. The Cr peak is due to the metallization process necessary for SEM observation. Structural analysis (Figure 1c) evidenced an amorphous halo at about 2θ = 25°–35° and a lack of any discrete diffraction peaks, confirming the completely amorphous nature of the starting glass.

SBA2 powders were then subjected to the ion-exchange process in different copper salt solutions. The pH of the solutions varied in different ranges, depending on the salt used and the concentration; after 1 h of contact with glass powders, the pH of the solutions increased due to the exchange between Na^+^ and Ca^2+^ ions from the glass and H^+^ ions from the solution (which is concurrent with the exchange with Cu^2+^), reaching higher values. In the case of copper nitrate solutions, the pH of the solution before the ion-exchange process varied between 3.9 and 4.7, and after the process the pH stabilized between 4.6 and 6.2. Regarding copper chloride solutions, the starting pH varied between 3.8 and 4.8, and after the process it reached values between 4.3 and 7.8. In the case of copper acetate solutions, the pH of the solution before the ion-exchange process varied between 5.5 and 5.7, and after the process the pH stabilized between 5.8 and 9.3. Finally, the pH of copper sulphate solutions changed between 4.3 and 5, and after the process reached values between 5.9 and 9.3, inclusive.

The pH of the solution can have an impact on the dissolution of the glass; nevertheless, it was reported that a strong increase of alkali dissolution was observed for bioactive glass with low silica content below pH 3 [43]—the pH of the used solution does not reach this value. Moreover, the ion-exchange process lasts only 1 h, whereas significant dissolution of bioactive glass with similar composition was observed by L. Björkvik *et al.* only after 7 or 14 days of treatment at pH near 2.

**Figure 2:**
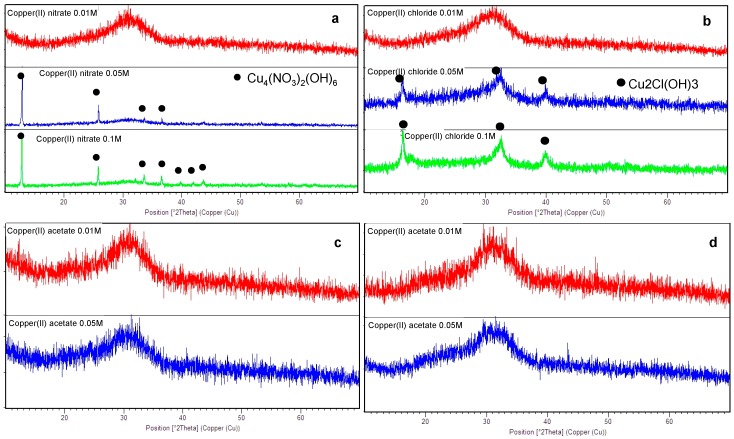
XRD analyses of SBA2 glass ion exchanged in (**a**) copper(II) nitrate; (**b**) copper(II) chloride; (**c**) copper(II) acetate; and (**d**) copper(II) sulphate solutions.

The structural characterization of ion-exchanged SBA2 glasses is reported in Figure 2. The ion-exchange in copper(II) nitrate 0.1 M and 0.05 M solutions caused the precipitation of a crystalline phase (Cu_4_(NO_3_)_2_(OH)_6_); on the contrary, the ion-exchange in a 0.01 M solution did not cause any additional phase precipitation, as confirmed by the XRD spectra, which revealed only the amorphous halo (Figure 2a). The same behaviour was observed for SBA2 powders ion-exchanged in copper(II) chloride solution: the formation of a Cu_2_Cl(OH)_3_ phase was observed after the ion-exchange in 0.1 M and 0.05 M solutions (Figure 2b), while any presence of crystallization peaks was estimated for SBA2 powders ion-exchanged in copper(II) acetate (Figure 2c) and sulphate solutions (both 0.05 M and 0.01 M) (Figure 2d).

**Figure 3:**
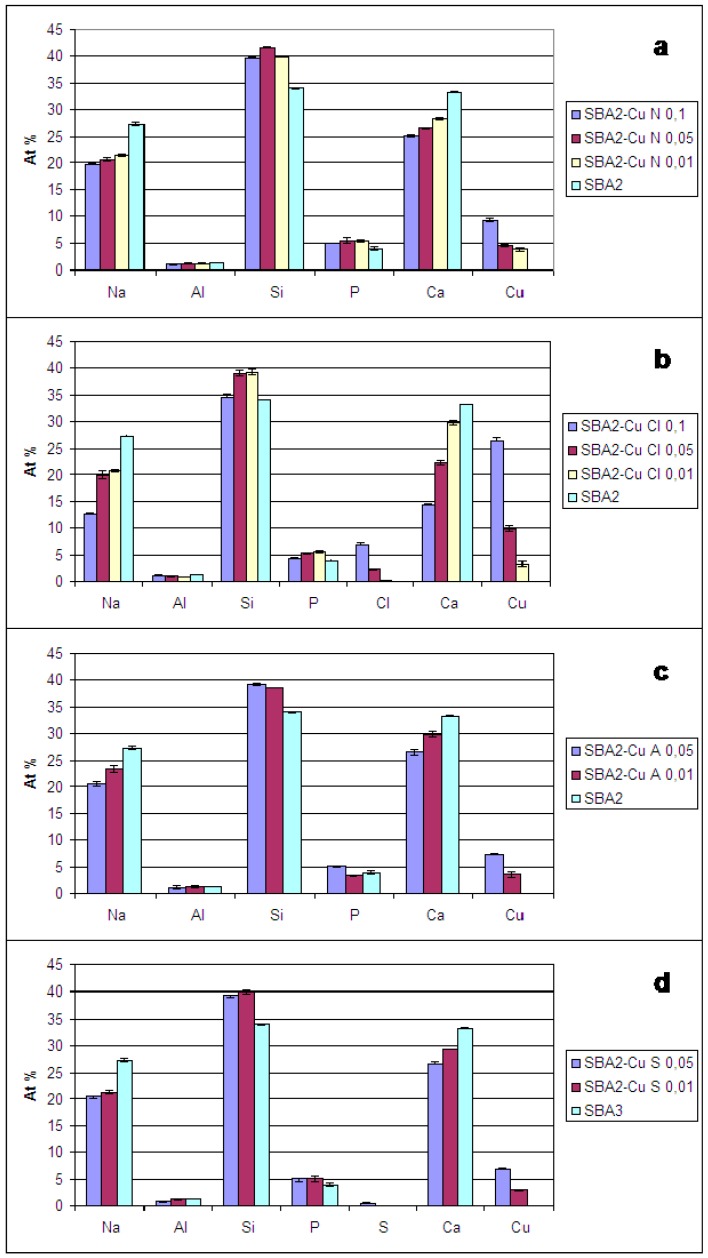
Energy Dispersion Spectrometry (EDS) analyses of SBA2 powders before ion-exchange after in (**a**) copper(II) nitrate (SBA2-Cu N); (**b**) copper(II) chloride (SBA2-Cu Cl); (**c**) copper(II) acetate (SBA2-Cu A); and (**d**) copper(II) sulphate solutions (SBA2-Cu S).

Figure 3 shows the results obtained from compositional analyses (EDS). As it can be observed, for all glasses, Cu replaced both sodium and calcium, since a decrease of both elements was observed in all glasses with respect to the estimated amount for SBA2 glass. The at % reduction of Na and Ca is a function of the ion-exchange conditions: the ion-exchange in more concentrated solutions leads to a greater decrease in the amount of these elements and, as a consequence, a higher amount of Cu was incorporated in the glass.

However, as observed in XRD analysis, for some ion-exchange conditions, a precipitation of Cu-containing crystalline phase occurred; then, in these cases, the amount of detected Cu includes both the Cu of the crystalline phase and that incorporated in the amorphous glass network. Table 2 displays the amount of Cu estimated by EDS analyses and the presence of Cu-containing crystalline phases individuated by XRD analysis for all exchanged glasses.

**Figure 4:**
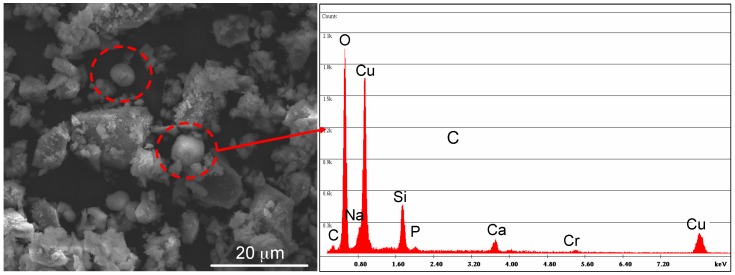
Morphological and compositional analyses of SBA2 ion exchanged in copper(II) acetate 0.05 M solution. Red circles evidenced a Cu-containing crystalline phase.

Since it was possible to introduce a higher amount of Cu in the glass by using copper(II) acetate as precursor, avoiding at the same time the precipitation of crystalline phases, glass powders exchanged in copper(II) acetate solutions were analysed by SEM-EDS. The morphological-compositional analysis (Figure 4) showed the presence of some Cu-containing crystals (evidenced with red circles in Figure 4) on SBA2 ion-exchanged in a copper(II) acetate 0.05 M solution, even if the presence of any additional crystalline phase was not detected by XRD analysis.

**Figure 5:**
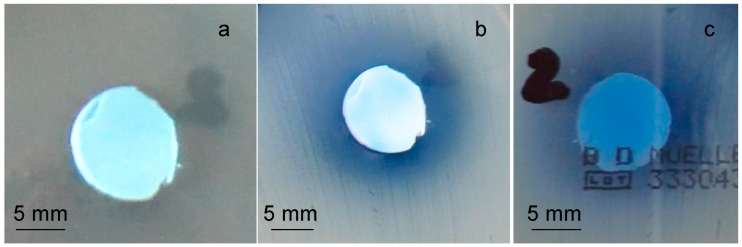
Inhibition halo of SBA2 glass ion exchanged in copper(II) acetate 0.01 M solution. (**a**) time zero; (**b**) front of the plate after 24 h of incubation; and (**c**) back of the plate after 24 h of incubation.

For this reason, the antibacterial test was performed on SBA2 powders ion-exchanged in copper(II) acetate 0.01 M solution; the obtained result is reported in Figure 5. As can be observed, the glass pellets were not able to create an inhibition halo towards *S. aureus* strain. The blue halo around the sample (Figure 5b,c) is due to Cu diffusion in the agar, but it is not an inhibition zone since bacterial colonies are visible inside the “blue area”. However, it seems that no bacteria proliferate under the sample (Figure 5c), revealing the ability of this material to affect bacterial adhesion.

### 3.2. SBA3 Characterization

**Figure 6:**
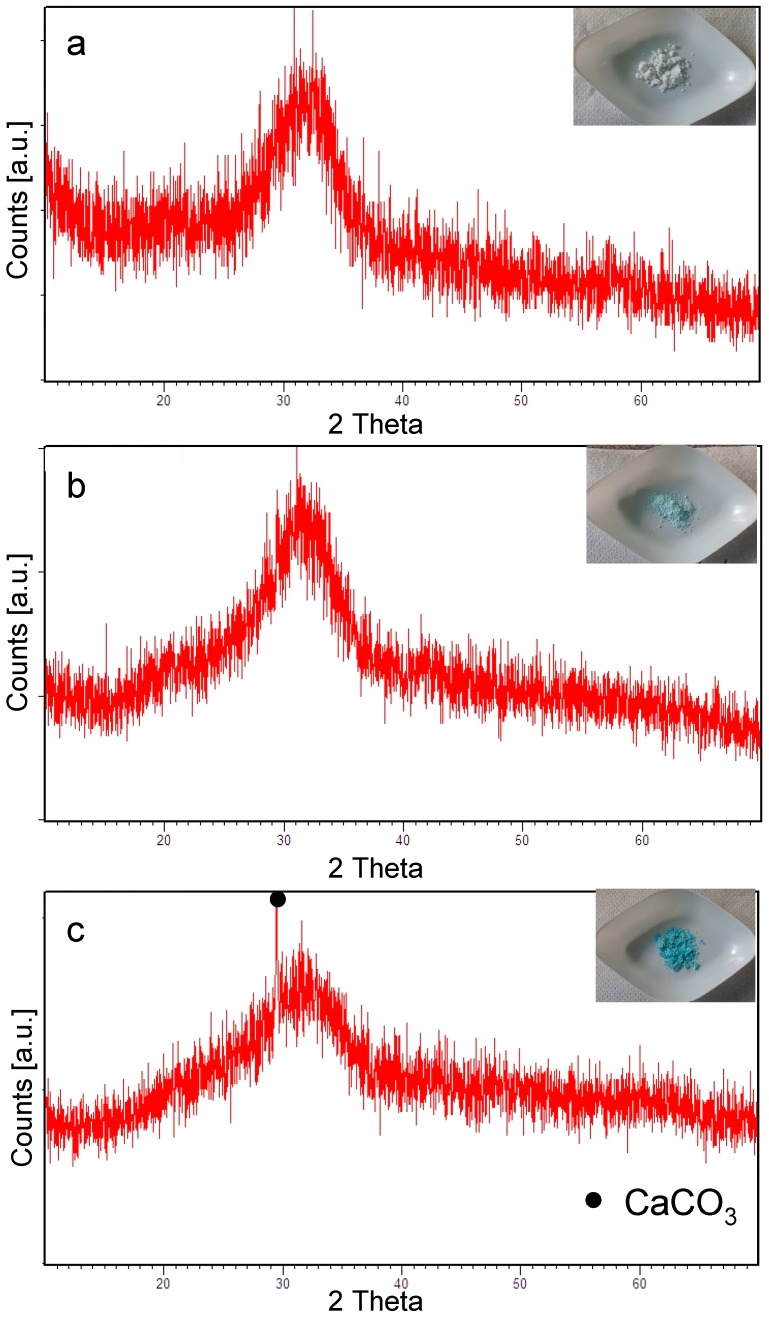
XRD analyses of SBA3 (**a**) before ion exchange; and after ion exchange in copper(II) acetate solution (**b**) 0.01 M and (**c**) 0.05 M.

On the basis of results obtained for SBA2 glass, SBA3 powders were subjected to ion-exchange process in an aqueous solution of copper(II) acetate 0.01 M and 0.05 M. Figure 6 shows the structural analyses of SBA3 before and after ion exchange processes. As can be noticed, SBA3 and SBA3-Cu0.01 are completely amorphous, while the SBA3-Cu0.05 spectrum shows a small peak at about 2 theta = 29.5°, which is ascribable to CaCO_3_; the formation of this phase could be occurring during the ion-exchange process.

**Figure 7:**
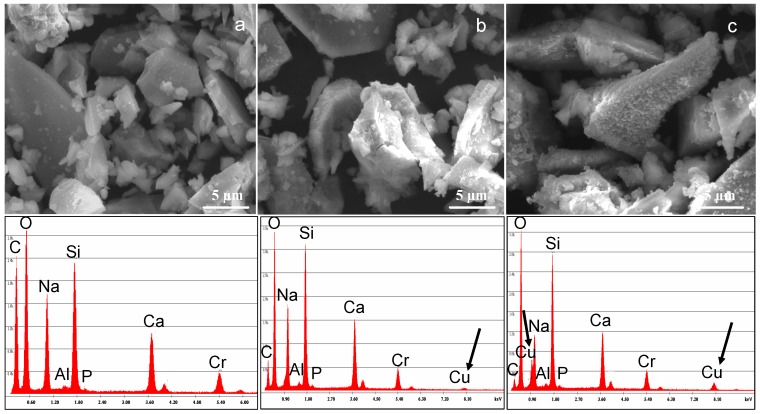
SEM-EDS analyses of (**a**) SBA3; (**b**) SBA3-Cu0.01; and (**c**) SBA3-Cu0.05.

Morphological characterization (Figure 7) evidenced an altered surface morphology (*i.e.*, a rougher surface) for ion-exchanged glasses in comparison to the pristine one, in particular for SBA3-Cu0.05. Compositional analyses confirmed the starting chemical composition of the SBA3 glass and the presence of Cu peaks in both SBA3-Cu0.01 and SBA3-Cu0.05 spectra. The amount of introduced Cu was 4.2 at % for SBA3-Cu0.01 and 14.2 at % for SBA3-Cu0.05; regarding SBA3-Cu0.01, the Cu ion-exchange occurred with sodium, while the ion exchange in a more concentrated solution (0.05 M) also led to an exchange with calcium (Table 3). The Cr peak visible in the EDS spectra is due to the metallization process necessary for SEM observation.

**Figure 8:**
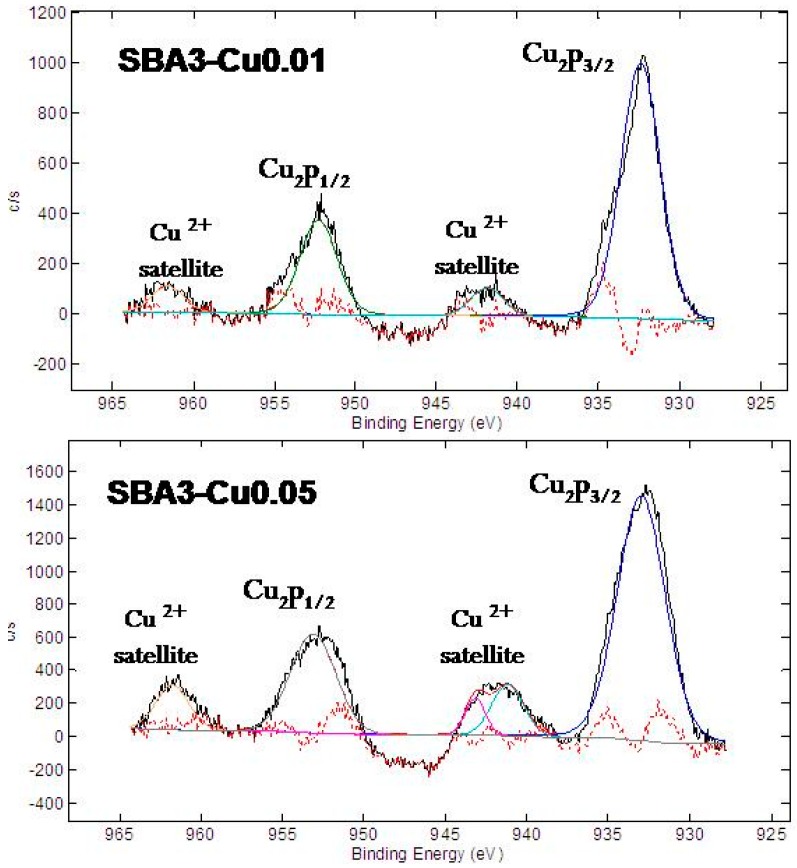
XPS detailed analyses of Cu 2p region of SBA3-Cu0.01 and SBA3-Cu0.05.

Table 4 and Figure 8 show the results of XPS measurements. The XPS quantitative analyses (Table 3) differ with respect to EDS analyses since EDS has a larger interaction volume then XPS (microns *vs.* nanometers); however, as evidenced with EDS measurements, the atomic percentage of Cu increased by increasing the concentration of ion-exchange solution; contemporaneously, sodium and calcium contents decreased. Al, B, and P were not detected.

In order to estimate the oxidation state of introduced Cu, a detailed analysis of the Cu (Cu 2p) region was performed. The XPS spectra show the peaks of Cu 2p_3/2_ and Cu 2p_1/2_ characteristic of Cu element; Cu(II) can be distinguished from Cu(I) and Cu^0^ since Cu(II) shows shake-up satellite features at about 943–941 eV and 961 eV [44]. These satellite peaks are well visible in SBA3-Cu0.01 and SBA3-Cu0.05 spectra, so the glass surface was enriched with Cu(II) ions.

**Figure 9:**
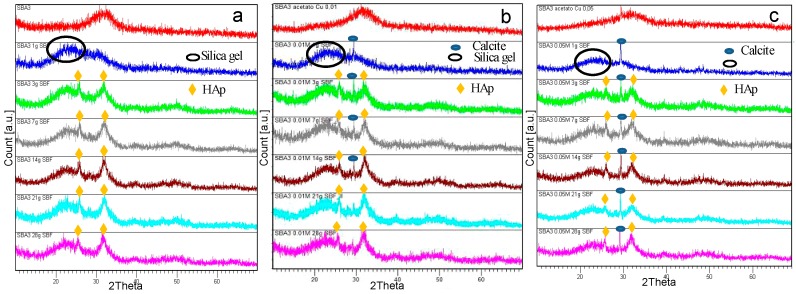
XRD spectra of SBA3 (**a**); SBA3-Cu0.01 (**b**); and SBA3-Cu0.05 (**c**) before and after immersion in simulated body fluid (SBF) for 1, 3, 7, 14, 21, and 28 days. HAp: Hydroxyapatite.

SBA3, SBA3-Cu0.01 and SBA3-Cu0.05 were subjected to *in vitro* bioactivity test to estimate the ability of SBA3 to induce the precipitation of hydroxyapatite and to verify the possible influence of Cu introduction and ion-exchange process on bioactivity. Figure 9 shows the XRD analyses performed on all three glasses before and after immersion in SBF solution up to 28 days. With regards to SBA3, after 1 day of SBF immersion, a broad halo between 20° and 25° ascribable to silica gel formation can be noticed. After 3 days of soaking, two peaks at 2 theta = 25.879° and 32.054° appeared; these peaks were attributed to hydroxyapatite (HAp) (ref. pattern 00-001-1008). The introduction of Cu did not influence the bioactivity mechanism: after 1 day of soaking in SBF, the characteristic halo of silica gel was also noticed in SBA3-Cu0.01 and SBA3-Cu0.05 spectra, together with a peak at about 2 theta = 29.452° ascribable to CaCO_3_ (ref. pattern 01-0830578). Calcite is a precursor of HAp and its precipitation has also been observed by other authors [45]. HAp precipitation was also clearly observed for Cu-doped glass after 3 days of immersion.

**Figure 10:**
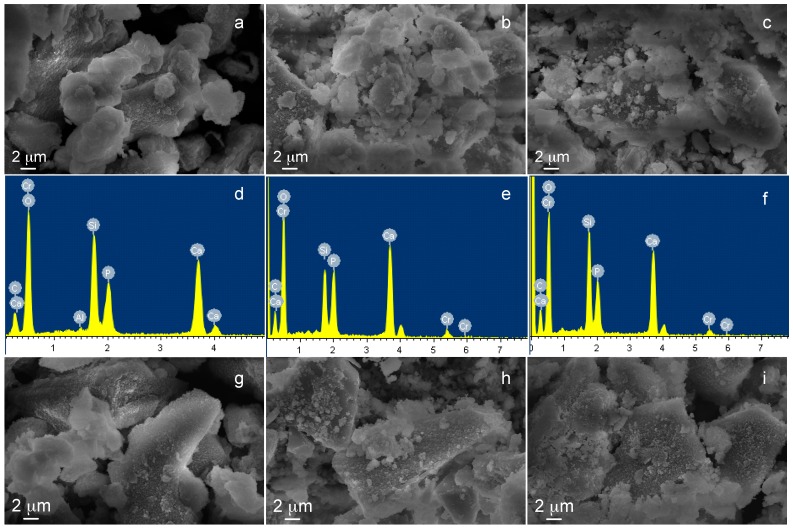
FESEM and EDS analysis of (**a**,**d**) SBA3; (**b**,**e**) SBA3-Cu0.01; and (**c**,**f**) SBA3-Cu0.05 after 21 days of immersion in SBF solution, and FESEM micrographs of (**g**) SBA3; (**h**) SBA3-Cu0.01; and (**i**) SBA3-Cu0.05 after 28 days of SBF treatment.

The results of FESEM-EDS analyses after *in vitro* bioactivity test are reported in Figure 10. The figure shows the sample morphology of SBA3, SBA3-Cu0.01, and SBA3-Cu0.05 after 21 (Figure 10a–c) and 28 (Figure 10g–i) days of immersion in SBF solution, and the compositional analysis (Figure 10d–f) of SBA3, SBA3-Cu0.01, and SBA3-Cu0.05 after 21 days of SBF treatment. All glasses showed a surface morphology clearly different with respect to the powders before SBF treatment, since they were covered by nanometric crystals rich in Ca and P. Micrographs after 21 and 28 days evidenced the presence of crystals grown on the glass powders; the local EDS analysis after 21 days of immersion shows very intense peaks of Ca and P. EDS analyses after 28 days were not reported since they are very similar to those after 21 days.

Then, the performed analyses indicated that the Cu introduced by ion-exchange process does not alter the bioactivity of pristine glass (SBA3), contrary to what has been observed by other authors [26,30] for Cu-doped glasses using sol-gel process or melt and quenching technique. In fact, Srivastava *et al.* reported that the bioactivity of melt-derived 45S5 glass doped with more than 1 wt % of CuO decreases, since Cu enhances the chemical durability of silicate glasses [30]. The same result was observed by Bejarano *et al.* for Cu-doped sol-gel glasses [26]—the presence of Cu in the glass decreased the ability to form surface crystalline apatite, since the incorporation of Cu generated a lower amount of Ca released and a competition between Cu^2+^ and Ca^2+^ ions in solution for the precipitation of phosphate species, which explains the lower bioactivity of the Cu-doped glasses. In the present work, Cu was introduced in very low amounts and confined in the outer surface layers. For these reasons, it is unlikely that its presence could strongly and negatively affect the release of the ions involved in the bioactivity mechanism from the doped glass (sodium and calcium in this case), as already observed by authors in a glass ion-exchanged with silver [46,47]. Moreover, as reported in Figure 7, the ion-exchange process caused an alteration of the surface morphology, with increasing roughness. As a consequence of this alteration, aside from the decrease of sodium and calcium ions (partially substituted by Cu), which should slow the bioactivity kinetics, a faster dissolution rate can be hypothesized [48] which, in turn, can positively affect the glass bioactivity, with a compensative effect toward sodium and calcium depletion.

**Figure 11:**
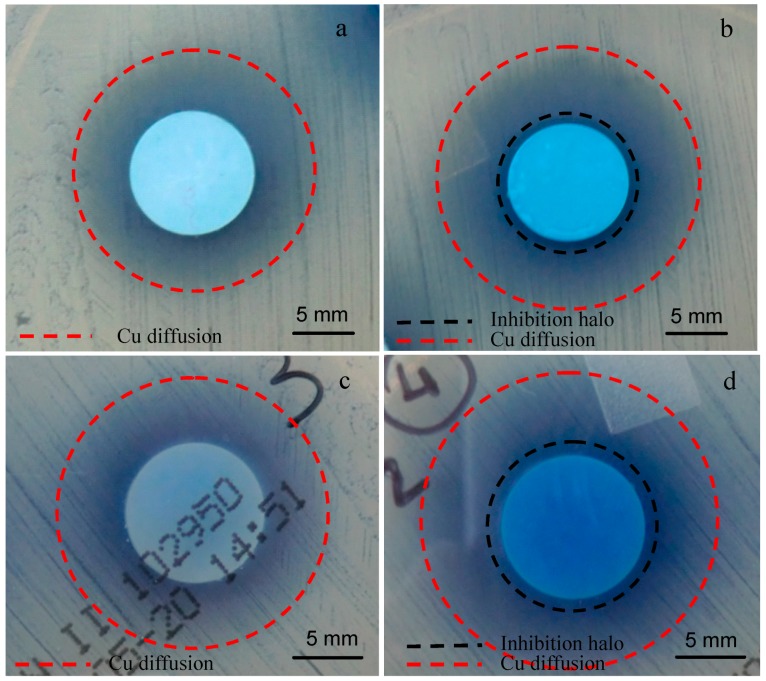
Inhibition halo test of (**a**,**c**) SBA3-Cu0.01; and (**b**,**d**) SBA3-Cu0.05; towards *S. aureus* strain, (**a**,**b**) front; and (**c**,**d**) back of the plate.

Antibacterial testing towards a strain of *S. aureus* is reported in Figure 11. As can be observed, Cu diffused from both samples in the agar plate (evidenced with red dotted line in Figure 11); SBA3-Cu0.01 was not able to produce an inhibition zone, while SBA3-Cu0.05 created a significant inhibition halo of about 1 mm. In addition, in this case, no bacteria proliferated under SBA3-Cu0.01 (Figure 11c), revealing the ability of this sample to avoid bacterial adhesion.

## 4. Conclusions

In this work, two bioactive glasses in the system SiO_2_, Na_2_O, CaO, P_2_O_5_, B_2_O_3_, Al_2_O_3_ (SBA2 and SBA3) have been synthesized by melt and quenching route and doped with Cu by ion-exchange in aqueous solutions. The effect of doping has been evaluated by morphological, compositional, and structural viewpoints, as well as in terms of bioactivity (HAp growth by soaking in SBF) and antibacterial properties (inhibition halo test).

Depending on both the glass composition and the ion-exchange conditions, Cu was introduced in the amorphous network as ionic species and as copper salts precipitates. By tailoring the glass reactivity (connected with the amount of monovalent modifying ions and the glass network degree of opening) and by proper selection of the exchange solution (in terms of typology of the copper salt and concentration), the process was optimized in order to enrich the glass surface with Cu(II) ions.

The ion-exchange process determined a modification of the morphology of the glass surface, inducing a higher roughness. The bioactivity of the glass, in terms of HAp growth by soaking in SBF, was not affected by the presence of Cu on the surface, contrary to what has been observed in literature for bioactive glasses doped with Cu in the whole bulk, where a competition between Ca^2+^ and Cu^2+^ ions has been observed in the first stages of the bioactivity mechanism. This difference from the literature can be explained by the relatively low amount of copper ions introduced by ion-exchange and their presence only in a very thin surface layer. This, in synergy with the increased surface roughness, which partially compensates for the eventual lower ion release from the glass, led to an unaltered ability of the glass to release ions involved in the bioactivity mechanism. Antibacterial testing on the optimized Cu-doped bioactive glasses revealed their ability to limit bacterial adhesion and proliferation.

Further investigations could be addressed to assess the biocompatibility of the glass modified with this new approach, as well as its ability to stimulate angiogenesis on human endothelial cell cultures.

## Figures and Tables

**Table 1:** Investigated conditions of ion-exchange.

Copper Salts	0.01 M	0.05 M	0.1 M
Copper(II) nitrate Cu(NO_3_)_2_·3H_2_O	x	x	x
Copper(II) chloride CuCl_2_·2H_2_O	x	x	x
Copper(II) acetate Cu(CH_3_COO)_2_·H_2_O	x	x	
Copper(II) sulphate CuSO_4_	x	x	

**Table 2:** Summary of Cu amount and presence of a crystalline phase detected by EDS and XRD analyses, respectively, for all ion-exchanged glasses.

Copper Solutions	at % Cu	Crystalline Phase
Copper(II) nitrate 0.1 M	9.2 ± 0.3	Yes
Copper(II) nitrate 0.05 M	4.5 ± 0.2	Yes
Copper(II) nitrate 0.01 M	3.7 ± 0.3	No
Copper(II) chloride 0.1 M	26.5 ± 0.6	Yes
Copper(II) chloride 0.05 M	9.9 ± 0.5	Yes
Copper(II) chloride 0.01 M	3.3 ± 0.5	No
Copper(II) acetate 0.05 M	7.4 ± 0.2	No
Copper(II) acetate 0.01 M	3.6 ± 0.5	No
Copper(II) sulphate 0.05 M	6.9 ± 0.1	No
Copper(II) sulphate 0.01 M	2.9 ± 0.1	No

**Table 3:** EDS analysis: atomic percentages of SBA3, SBA3-Cu0.01, and SBA3-Cu0.05.

EDS	SBA3	SBA3-Cu0.01	SBA3-Cu0.05
Elements	at %	at %	at %
Na	32.1	27.7	22.7
Al	1.6	1.1	2
Si	38.9	39.5	37.9
P	0.6	0.7	0.7
Ca	26.9	26.9	22.5
Cu	0	4.2	14.1

**Table 4:** Atomic percentages of elements obtained from XPS analyses of SBA3, SBA3-Cu0.01 and SBA3-Cu0.05.

XPS-Elements	SBA3	SBA3-Cu0.01	SBA3-Cu0.05
Na	56.9	38.2	29.4
Si	25.8	43.3	46.6
Ca	17.3	11.6	7.0
Cu	0.00	6.9	17.0
